# The association between physical activity and perceived stress among adolescents: the serial mediating roles of social-emotional competence and prosocial behavior

**DOI:** 10.3389/fpsyt.2026.1947638

**Published:** 2026-09-16

**Authors:** Jiaqi Qu, Chuxi Shang, Xuyao Ding, Shanjie Liu, Chaofan Shi, Bingao Chen, Yingbo Zhu

**Affiliations:** 1 School of Physical Education, Henan University, Kaifeng, China; 2 School of Physical Education and Sports, Central China Normal University, Wuhan, China; 3 Faculty of Physical Education and Physiotherapy, Vrije Universiteit Brussel, Brussel, Belgium; 4 School of Physical Education, Yunnan Normal University, Kunming, Yunnan, China

**Keywords:** adolescents, perceived stress, physical activity, prosocial behavior, social-emotional competence

## Abstract

**Background:**

Adolescence is a critical developmental period characterized by heightened vulnerability to stress. Stressors such as academic demands, interpersonal relationships, and the family environment may intensify perceived stress (PS). Physical activity (PA) has been associated with adolescents’ appraisal of and coping with stressful events, potentially in relation to psychosocial resources. This study examined the association between PA and PS and investigated the potential roles of social-emotional competence (SEC) and prosocial behavior (PB).

**Methods:**

PA, SEC, PB, and PS were assessed using the Physical Activity Rating Scale-3 (PARS-3), the Social-Emotional Competence Questionnaire, the Prosocial Tendencies Measure, and the 14-item Perceived Stress Scale (PSS-14), respectively. Descriptive statistics, Pearson correlation analysis, and hierarchical multiple regression analysis were conducted using SPSS 27.0. PROCESS 4.1 Model 6 was used to test the independent and serial mediating effects of SEC and PB.

**Results:**

(1) PA was significantly negatively associated with PS (*β* = −0.398, *p* < 0.001). This association remained significant after SEC and PB were included (*β* = −0.216, *p* < 0.001). (2) The indirect association between PA and PS involving SEC was statistically significant (*b* = −0.025, 95% CI [−0.030, −0.020]). (3) The indirect association between PA and PS involving PB was statistically significant (*b* = −0.016, 95% CI [−0.020, −0.013]). (4) The serial indirect association between PA and PS involving SEC and PB was statistically significant (*b* = −0.009, 95% CI [−0.011, −0.007]).

**Conclusion:**

PA was associated with lower PS among adolescents, and the indirect associations were consistent with the prespecified ordering of SEC and PB. These findings may inform future research on how team sports and sport-based public-service activities relate to adolescents’ developmental resources, coping, and PS.

## 1 Introduction

Amid rapid social transformation and intensifying educational competition, adolescents are chronically exposed to multiple stressors, including academic competition, interpersonal conflict, and adjustment demands. The resulting accumulation of tension and perceived uncontrollability can seriously impede mental health development (1, 2). In 2025, the World Health Organization again emphasized in its report on adolescent mental health that persistently high stress is an important risk factor for mental disorders among adolescents (3). Perceived stress (PS) refers to an individual’s subjective psychological response when coping with threatening or challenging events and reflects an important aspect of mental health (4). PS is closely associated not only with adolescents’ academic stress and quality of life but also with problematic behaviors, such as internet addiction and substance misuse, as well as the risk of suicidal ideation and suicidal behavior (5–7). Thus, the adverse consequences of PS extend from diminished quality of life to high-risk psychological and behavioral problems, making it a major psychological risk factor that threatens adolescents’ overall well-being and developmental resources. Identifying protective psychological resources may therefore provide empirical support for improving school mental health services and developing public health strategies to prevent and manage adolescent mental health problems.

Conservation of resources (COR) theory proposes that the protective psychological resources possessed by adolescents facilitate coping with external stressors, reduce psychological distress, and may attenuate the adverse effects of negative events on PS (8, 9). Previous research has begun to examine the relationships between protective psychological factors and PS. For example, social-emotional competence (SEC) may enhance social adjustment and reduce the adverse effects of negative interpersonal events on PS (10, 11). Prosocial behavior (PB) may reduce the depletion associated with stress appraisal through supportive peer relationships and social support, thereby lowering adolescents’ PS (12, 13). Behavioral indicators have recently attracted increasing attention because they are externally observable and may regulate both physiological and psychological processes. Physical activity (PA) may alleviate physiological stress responses associated with stress symptoms and improve cognitive functions such as executive function and working memory (5, 14, 15). Regular exercise may also reduce PS by decreasing psychological vulnerability and improving stress-related emotional states (16, 17). PA may therefore represent an important protective factor against PS; however, empirical evidence concerning the association between PA and PS among adolescents remains limited.

Accordingly, this study constructed a serial mediation model to examine the sequential mediating effects of SEC and PB on the association between PA and PS. By clarifying the pathways through which PA is associated with adolescent mental health, the study aimed to provide empirical support for school-based stress interventions and mental health education.

### 1.1 Physical activity and perceived stress

Given the physiological sensitivity that characterizes adolescence, PA may alleviate stress responses through two pathways—internal physiological regulation and external social resources—thereby reducing the likelihood that negative events are appraised as stressful. At the physiological level, regular PA may discharge stress-induced hyperarousal, regulate activation of the sympathetic nervous system and the hypothalamic-pituitary-adrenal axis, and reduce persistent tension and perceived uncontrollability when adolescents encounter negative events such as academic pressure and interpersonal conflict (18). At the social-resource level, and consistent with the need-to-belong framework, group-based PA may improve adolescents’ cognitive appraisal of stressful real-life situations and thereby reduce PS. Specifically, group exercise provides stable opportunities for peer interaction and facilitates the development of reliable peer-support networks. Peer support can provide emotional reassurance and coping resources that buffer the sense of threat arising from academic comparison and negative interpersonal evaluation (19–21).

No consensus has yet been reached regarding the pathways through which PA buffers PS. A nationwide survey in South Korea found that the negative association between PA and PS was present only among adolescents in medium-sized cities, suggesting possible regional variation (22). Moreover, some cross-sectional studies have relied primarily on questionnaire data to examine the correlation between the two variables and have provided limited theoretical explanation of the underlying mechanisms (23). Existing evidence on the stress-buffering role of PA among adolescents has also been based largely on samples outside China. For example, Wright et al. identified a mediating role of self-esteem in the association between PA and PS among older adolescents in the United Kingdom (24). Overall, research on the association between PA and PS among Chinese adolescents remains insufficient. Further investigation of the mechanisms linking PA to adolescent PS is therefore warranted.

### 1.2 The mediating role of SEC in the association between PA and PS

SEC refers to the capacity to understand and regulate emotions in complex social situations and to establish and maintain positive social relationships (25). It constitutes an important positive psychological resource during adolescence and may reduce PS while enhancing adaptation to stressful situations (9, 26). SEC integrates emotional and social capacities. Core competencies such as emotion management and interpersonal communication may help adolescents coordinate emotional responses with interpersonal demands in stressful contexts and develop adaptive coping strategies (27, 28). Empirical evidence further indicates that individuals with higher SEC are more likely to use adaptive emotion-regulation strategies autonomously, thereby reducing the loss of psychological resources caused by negative emotions (29). Higher SEC may therefore reduce psychological resource depletion under stress and be associated with lower PS.

Through its contextual, interactive, and experiential characteristics, PA provides adolescents with sustained opportunities for socialization and for developing emotion management, empathy, and cooperation, making it an important context for the development of SEC (30). Within situated classroom practice, PA can provide continuing opportunities for interpersonal interaction and social learning through collaborative tasks and authentic communication (31, 32). Particularly in team sports, ongoing peer interaction and relational support may foster executive function and emotion regulation and, in turn, promote adolescents’ psychological and social adjustment (33–35). In a four-year longitudinal study of 2,278 children, Bedard et al. (36) found that both school-based and extracurricular sport participation were associated with small improvements in children’s self-perceived social competence, with comparable associations among boys and girls (36). Accordingly, this study treated SEC as a key adaptive capacity and examined its mediating role in the association between PA and PS, with the aim of informing efforts to promote adolescent mental health and social development.

### 1.3 The mediating role of PB in the association between PA and PS

PB refers to behaviors displayed in social and interpersonal interactions that conform to social norms and benefit other individuals or groups (37). Unlike psychological resources that operate primarily through intrapersonal regulation, PB is associated with more adaptive stress coping and lower PS. It may strengthen belonging and self-worth by promoting positive interpersonal connections and social support (38, 39). More specifically, observable helping and supportive actions may facilitate supportive social interactions and buffer stress and loneliness by strengthening social connectedness (40, 41). Consistent with this mechanism, Shi et al. (42) found that adolescents with high PB reported significantly lower PS than those with low PB, with perceived social support and self-esteem serving as mediators (42). These findings suggest that PB may be associated with lower PS.

As an important context for fostering PB among adolescents, PA may positively guide behavioral development (43). From a biopsychosocial perspective, the association between PA and PB can be understood across three interrelated domains: neurophysiology, psychological cognition, and social interaction. Improvements in neuroplasticity, executive control, and emotional processing associated with regular exercise may provide a neurocognitive basis for inhibiting self-interested impulses, considering others’ interests, and making prosocial decisions (44, 45). Positive experiences and feedback during exercise may foster positive self-perceptions and thereby increase tendencies toward cooperation and helping (37). Goal interdependence, role coordination, and conflict negotiation in team activities may help adolescents understand others’ needs, develop positive empathy, and translate that empathy into helping, sharing, and cooperation (46, 47). Therefore, PB may mediate the association between PA and adolescent PS.

### 1.4 The serial mediating roles of SEC and PB in the association between PA and PS

SEC and PB are closely connected positive developmental resources during adolescence (48). SEC encompasses the cognitive, emotional, and interpersonal capacities needed to understand others’ emotional states and regulate one’s own responses. Empathy and emotion recognition help adolescents identify others’ needs, whereas emotion regulation and self-control help manage personal distress and inhibit self-focused impulses. Together, these capacities support the translation of prosocial concern into contextually appropriate helping, sharing, and cooperative behavior (49–51). Consistent with this account, meta-analytic evidence shows that school-based social-emotional learning interventions improve both social-emotional skills and behavioral outcomes (52). Longitudinal studies further indicate that self-regulation predicts later PB across home and peer contexts and that earlier emotional self-regulation is associated with subsequent PB among adolescents (53–55). SEC may therefore function as an internal developmental resource that enables adolescents to express social understanding and emotional concern through observable prosocial actions.

These two protective resources may constitute a key sequential pathway linking PA to adolescent PS. From the perspective of COR theory, longitudinal cross-lagged evidence suggests that physical exercise improves internal psychological attributes such as emotion regulation and self-control, thereby laying a foundation for the development of SEC (56). Meanwhile, the face-to-face interactions afforded by PA may transform internal psychological strengths into positive social functioning, expand peer-support networks, and further promote PB (57). PA may thus cultivate internal social-emotional resources, including empathy and emotion regulation, which may subsequently facilitate helping and inclusive PB and ultimately buffer PS (58, 59). Accordingly, this study incorporated SEC and PB into a single analytic model to examine their mediating roles and sequential association between PA and adolescent PS, thereby providing a theoretical basis for promoting adolescent mental health and social development.

### 1.5 Hypotheses

This study examined whether SEC and PB serially mediated the association between PA and PS among school-attending adolescents recruited from six secondary schools in Shandong and Henan Provinces, China. Although previous studies have reported separate associations among PA, SEC, PB, and PS, most have focused on bivariate relationships, and systematic analyses within a unified theoretical framework remain scarce, particularly among adolescents. According to COR theory, PA can help individuals acquire psychological and social resources, while SEC may promote PB and enhance stress coping through positive interpersonal interaction and social support. Based on the existing literature, four hypotheses were proposed (**Figure 1**).

**Figure 1:**
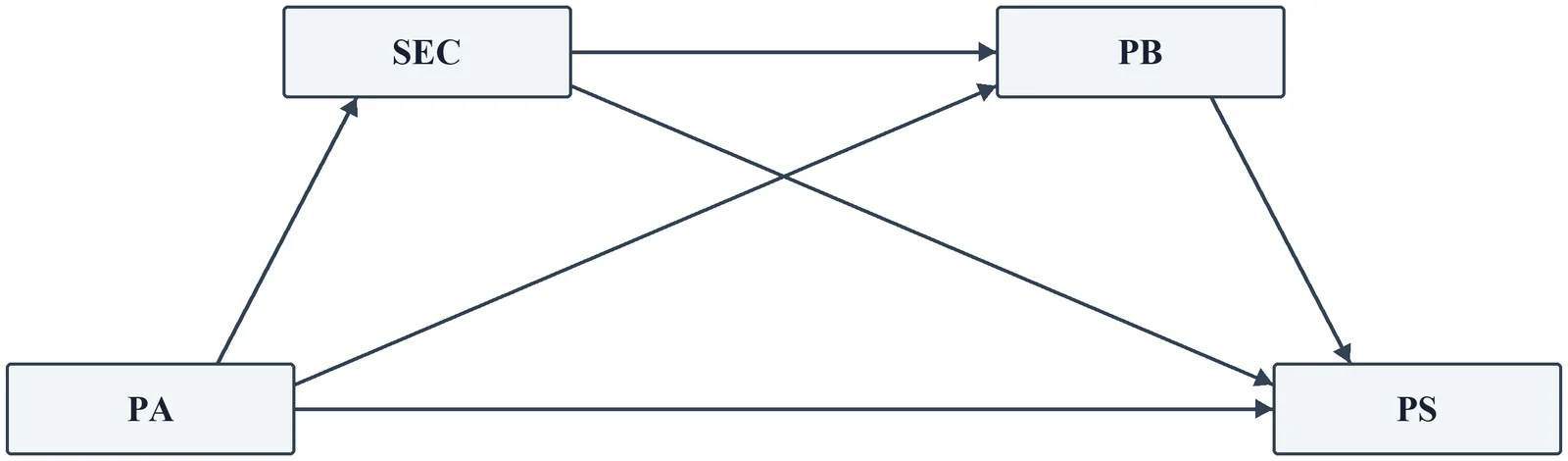
Theoretical model. PA = physical activity; SEC = social-emotional competence; PB = prosocial behavior; PS = perceived stress.

H1: PA is significantly negatively associated with PS among adolescents.

H2: SEC independently mediates the association between PA and PS among adolescents.

H3: PB independently mediates the association between PA and PS among adolescents.

H4: SEC and PB serially mediate the association between PA and PS among adolescents.

## 2 Methods

### 2.1 Participants

Participants were students from six secondary schools in Shandong and Henan Provinces, China. The final sample comprised 2,873 adolescents, including 1,429 boys (49.74%) and 1,444 girls (50.26%). Participants were 12–16 years old (*M* = 13.53, *SD* = 0.95). The sample included 984 seventh-grade students (34.25%), 980 eighth-grade students (34.11%), and 909 ninth-grade students (31.64%). Participant characteristics are presented in **Table 1**.

**Table 1:** Demographic characteristics of the participants (N = 2,873).

Demographic variables		Number	Proportion (%)
Age (years)	13.53 ± 0.95	2,873	100.0%
Sex	Male	1,429	49.7%
	Female	1,444	50.3%
Grade level	Seventh Grade	984	34.2%
	Eighth Grade	980	34.1%
	Ninth Grade	909	31.6%
Place of birth	Rural	1,271	44.2%
	Urban	1,602	55.8%
Only child	Yes	1,300	45.2%
	No	1,573	54.8%
Boarding student	Yes	896	31.2%
	No	1,977	68.8%

The inclusion criteria were as follows: age 12–16 years; ability to understand and complete the questionnaire independently; guardian informed consent; and adolescent assent. Individuals who did not provide informed consent or assent, could not complete the questionnaire independently, or failed to meet the prespecified questionnaire-quality criteria were excluded. See **Figure 2** for the specific screening process.

**Figure 2:**
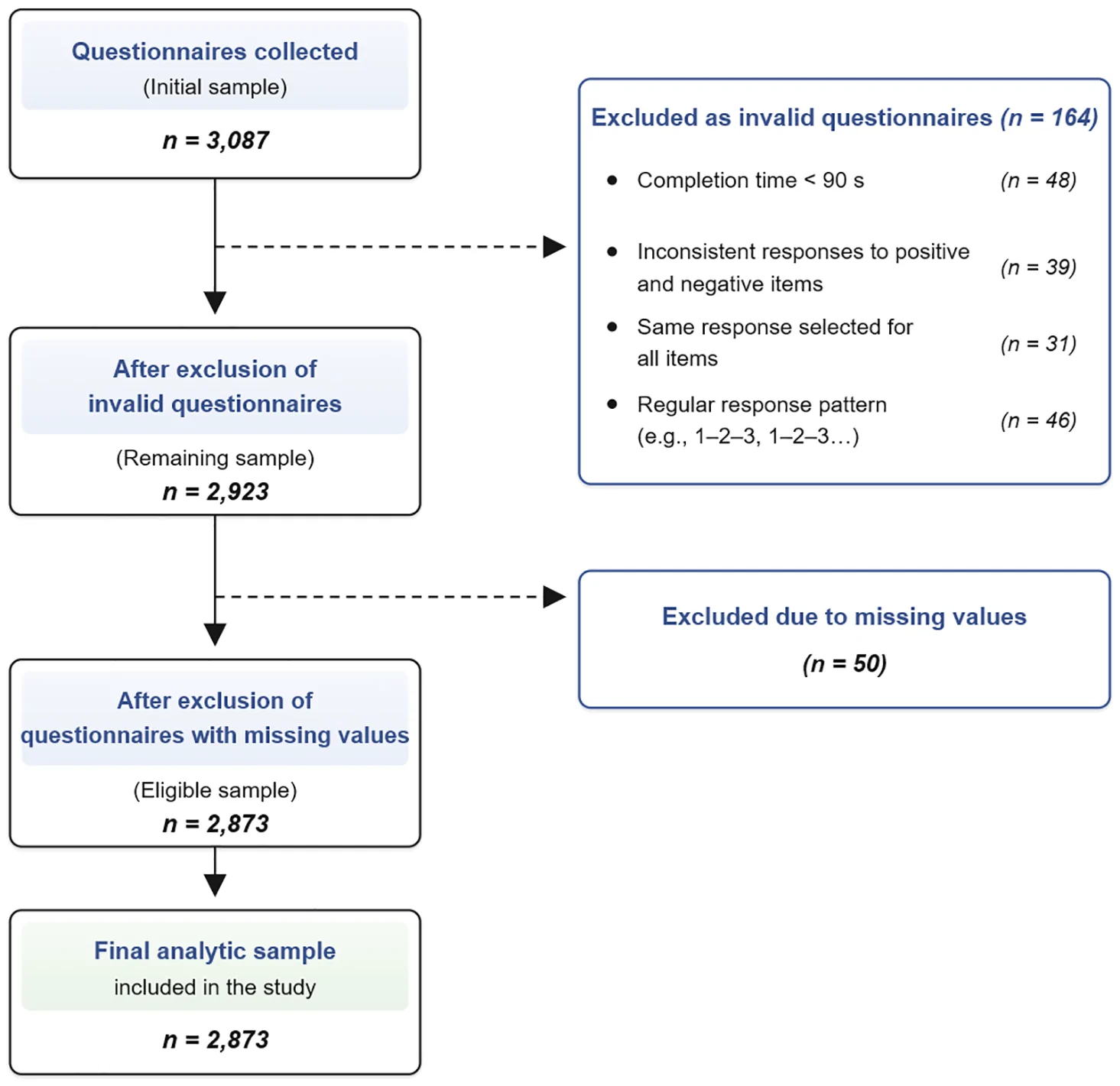
Participant screening flowchart.

### 2.2 Procedure

The study was conducted in Shandong and Henan Provinces, China, from June to July 2026. Six secondary schools were selected through convenience sampling, after which classes within each school were selected using cluster random sampling. Eligible adolescents in the selected classes were invited to participate. Before data collection, the researchers explained the study purpose, voluntary nature of participation, anonymity, and confidentiality procedures to school personnel, guardians, and adolescents, and obtained guardian informed consent and adolescent assent. The study protocol was approved by the Biomedical Research Subcommittee of Henan University (approval no. HUSOM2026-749).

Questionnaires were administered online through the Wenjuanxing platform. A survey link or QR code was distributed to the selected classes, and adolescents completed the questionnaire independently following standardized instructions. No names or other direct personal identifiers were collected, and participants could withdraw at any time before submission. After data collection, responses were screened using prespecified criteria for completion time, duplicate submissions, patterned responding, and logical consistency. Invalid questionnaires were excluded before the final analytic dataset was established.

### 2.3 Measures

#### 2.3.1 Physical activity

The Physical Activity Rating Scale-3 (PARS-3), developed by Liang Deqing, was used to assess adolescents’ PA (60). The scale provides a composite assessment across three dimensions: intensity, frequency, and duration. Intensity and frequency are each rated on a 1–5 scale, whereas duration is rated from 0 to 4. The total PA score is calculated as the product of the three dimension scores (range: 0–100), and PA is classified as low (≤19), moderate (20–42), or high (≥43). The PARS-3 has been widely used in related research (61). In the present study, Cronbach’s α was 0.783, indicating acceptable internal consistency.

#### 2.3.2 Social-emotional competence

SEC was assessed using the Social-Emotional Competence Questionnaire developed by the United Nations Children’s Fund–Ministry of Education Social and Emotional Learning project team (62). Adapted for the Chinese context on the basis of the CASEL framework, the 30-item questionnaire comprises six dimensions: self-cognition, self-awareness, cognition of others, management of others, collective cognition, and collective management. Items are rated on a 5-point Likert scale (1 = completely untrue of me; 5 = completely true of me), with higher total scores indicating greater SEC. Its applicability among adolescents has received empirical support (63). Cronbach’s α was 0.867 in the present study, indicating good internal consistency.

#### 2.3.3 Prosocial behavior

PB was assessed using the Chinese version of the Prosocial Tendencies Measure, originally developed by Carlo and Randall (64) and revised by. (65). The 26-item scale comprises six dimensions: public, altruistic, anonymous, emotional, compliant, and dire PB. Items are rated on a 5-point Likert scale, with higher total scores indicating a stronger tendency toward PB. The measure has demonstrated good applicability among adolescents (66). Cronbach’s α was 0.857 in the present study, indicating good internal consistency.

#### 2.3.4 Perceived stress

PS during the previous month was assessed using the 14-item Perceived Stress Scale (PSS-14), developed by (67) and revised by (68). The scale comprises two dimensions, tension and perceived uncontrollability. Items are rated on a 5-point scale (1 = never; 5 = always) and summed to yield a total score ranging from 14 to 70, with higher scores indicating greater PS. The measure has been validated in related research (4). Cronbach’s α was 0.807 in the present study, indicating good internal consistency.

### 2.4 Statistical analysis

Data were analyzed using SPSS 27.0. Harman’s single-factor test was used to assess common method bias. Descriptive analyses were conducted to characterize the score distributions, and Pearson correlation analysis was used to examine associations among the study variables. Hierarchical multiple linear regression models were constructed to test the main effects underlying the proposed serial mediation model. The PROCESS macro developed by Hayes (69) was then used to test the serial mediating effects. Indirect effects were evaluated using bias-corrected bootstrapping with 5,000 resamples and 95% confidence intervals (CIs).

## 3 Results

### 3.1 Common method bias

Because all variables were assessed by self-report, Harman’s single-factor test was conducted to evaluate potential common method bias (70). An unrotated principal component analysis of all 73 scored items extracted 15 factors with eigenvalues greater than 1, which together explained 56.448% of the total variance. The first factor accounted for 15.221% of the variance, below the 40% screening criterion. To provide a more stringent assessment, latent common-method-factor models were additionally estimated. The method factors accounted for only 1.99%–2.41% of item variance on average, and substantive factor loadings remained stable. These findings indicated that common-method variance was limited and did not appear to substantially distort the measurement structure (Supplementary Results).

### 3.2 Descriptive statistics and correlation analysis

**Table 2** presents the means, standard deviations, and correlations for PS, PA, SEC, and PB. PA was categorized as low (≤19), moderate (20–42), or high (≥43). Of the participants, 45.0% had low PA, 28.3% had moderate PA, and 26.7% had high PA. The mean scores were 27.49 ± 6.67 for PS, 30.18 ± 24.56 for PA, 123.62 ± 11.81 for SEC, and 90.23 ± 12.00 for PB.

**Table 2:** Means, standard deviations, and correlations among the study variables (*N* = 2,873).

Variable	*M*	*SD*	1	2	3	4
1. Perceived stress	27.49	6.67	1	—	—	—
2. Physical activity	30.18	24.56	-0.418***	1	—	—
3. Social-emotional competence	123.62	11.81	-0.419***	0.452***	1	—
4. Prosocial behavior	90.23	12.00	-0.421***	0.461***	0.501***	1

*M* = mean; *SD* = standard deviation. *** *p* < 0.001 (two-tailed).

PA was significantly negatively correlated with PS (*r* = −0.418, *p* < 0.001), as were SEC (*r* = −0.419, *p* < 0.001) and PB (*r* = −0.421, *p* < 0.001). PA was significantly positively correlated with both SEC (*r* = 0.452, *p* < 0.001) and PB (*r* = 0.461, *p* < 0.001), and SEC was significantly positively correlated with PB (*r* = 0.501, *p* < 0.001). These associations provided a statistical basis for the subsequent serial mediation analysis.

### 3.3 Hierarchical regression analysis

Hierarchical multiple regression analysis was performed to examine whether PA, SEC, and PB predicted PS (**Table 3**). Multicollinearity diagnostics showed that variance inflation factor (VIF) values for the focal predictors ranged from 1.028 to 1.479 and tolerance values ranged from 0.676 to 0.973, indicating no substantial multicollinearity and supporting the use of regression and mediation analyses.

**Table 3:** Results of the hierarchical regression analyses.

Variables		Dependent variable:PS								SEC		PB	
		Model 1		Model 2		Model 3		Model 4		Model 5		Model 6	
		β	t	β	t	β	t	β	t	β	t	β	t
Control variables	Gender	0.032	1.76	0.029	1.72	0.020	1.22	0.014	0.86	-0.033*	-1.97	-0.030	-1.94
	Age	0.051*	2.57	0.042*	2.33	0.038*	2.19	0.032	1.87	-0.015	-0.82	-0.030	-1.82
	Grade level	-0.009	-0.44	-0.007	-0.36	-0.001	-0.08	0.004	0.26	0.019	1.04	0.028	1.70
	Place of birth	-0.055**	-3.00	-0.030	-1.80	-0.025	-1.55	-0.020	-1.23	0.018	1.11	0.027	1.74
	Only-child status	-0.073***	-3.99	-0.038*	-2.26	-0.031	-1.94	-0.028	-1.74	0.024	1.45	0.018	1.20
	Boarding status	0.157***	8.55	0.108***	6.34	0.089***	5.46	0.084***	5.21	-0.065***	-3.90	-0.028	-1.81
Independent variable	PA	—	—	-0.398***	-23.36	-0.275***	-15.08	-0.216***	-11.51	0.440***	26.14	0.289***	16.69
Mediating variable	SEC	—	—	—	—	-0.280***	-15.40	-0.206***	-10.73	—	—	0.363***	21.00
	PB	—	—	—	—	—	—	-0.205***	-10.65	—	—	—	—
Model fit	R	0.191		0.437		0.502		0.530		0.459		0.569	
	R²	0.036		0.191		0.252		0.281		0.210		0.324	
	Adjusted R²	0.034		0.189		0.250		0.279		0.209		0.322	
	ΔR²	0.036		0.154		0.062		0.028		0.188		0.300	
	F	18.03***		96.35***		120.92***		124.29***		109.10***		171.62***	

Standardized regression coefficients (β) and their corresponding t values are reported for all predictor variables; intercepts are omitted. PA = physical activity; SEC = social–emotional competence; PB = prosocial behavior; PS = perceived stress. p <.05, p <.01, p <.001.

Model 1 included sex, age, grade level, place of birth, only-child status, and boarding status as demographic covariates. After adjustment for these covariates, Model 2 showed that PA significantly negatively predicted PS (*β* = −0.398, *p* < 0.001). After SEC was added in Model 3, both PA (*β* = −0.275, *p* < 0.001) and SEC (*β* = −0.280, *p* < 0.001) significantly negatively predicted PS. After PB was added in Model 4, PA (*β* = −0.216, *p* < 0.001), SEC (*β* = −0.206, *p* < 0.001), and PB (*β* = −0.205, *p* < 0.001) each significantly negatively predicted PS.

In Model 5, with SEC as the dependent variable, PA significantly positively predicted SEC (*β* = 0.440, *p* < 0.001). In Model 6, with PB as the dependent variable, both PA (*β* = 0.289, *p* < 0.001) and SEC (*β* = 0.363, *p* < 0.001) significantly positively predicted PB. These findings were consistent with the direction of the proposed serial pathway, PA → SEC → PB → PS.

### 3.4 Mediation analysis

The serial mediation estimates are presented in **Table 4** and **Figure 3**. The estimated total association between PA and PS was statistically significant (b = −0.1082, 95% CI [−0.117, −0.099]). After SEC and PB were included in the model, the estimated direct association between PA and PS remained statistically significant (b = −0.0586, 95% CI [−0.068, −0.049]) and accounted for 54.14% of the total association. The estimated total indirect association was −0.0496 (95% CI [−0.056, −0.044]) and accounted for 45.86% of the total association. The bootstrap confidence interval for each indirect association excluded zero, providing statistical support for all three indirect associations.

**Table 4:** Serial mediation effects and effect proportions.

Path	Effect (b)	SE	95% CI		Proportion (%)
			Lower bound	Upper bound	
Total effect	-0.1082	0.004	-0.117	-0.099	—
Direct effect	-0.0586	0.005	-0.068	-0.049	54.14%
Total indirect effect	-0.0496	0.003	-0.056	-0.044	45.86%
Ind1: PA→SEC→PS	-0.0246	0.003	-0.030	-0.020	49.53%
Ind2: PA→PB→PS	-0.0161	0.002	-0.020	-0.013	32.52%
Ind3: PA→SEC→PB→PS	-0.0089	0.001	-0.011	-0.007	17.95%

Covariate-adjusted total, direct, and indirect effects are reported as unstandardized coefficients (b). The 95% confidence intervals for the indirect effects were based on 5,000 bootstrap resamples.

**Figure 3:**
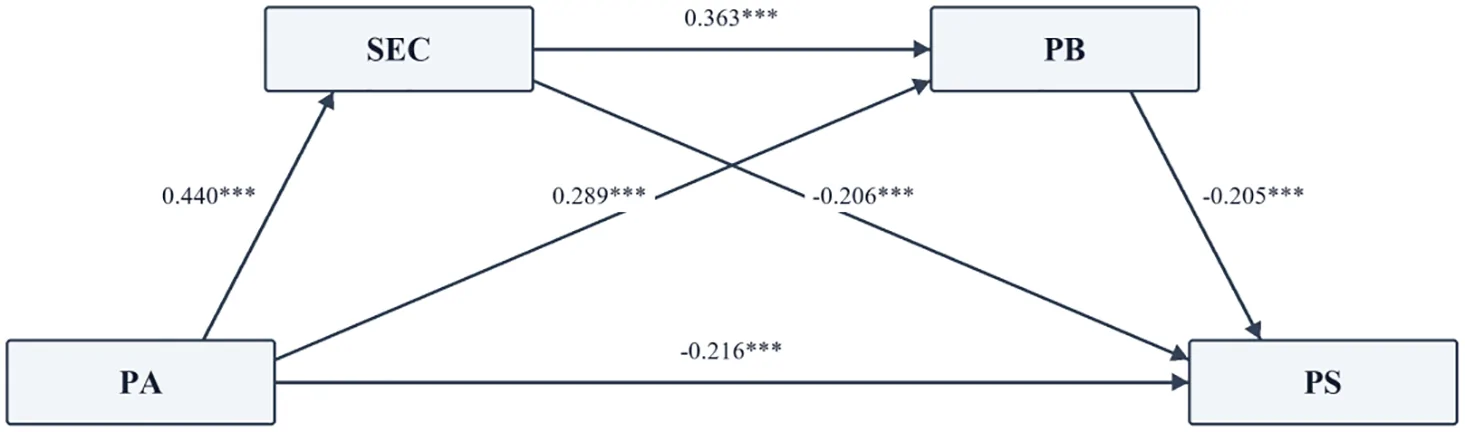
Serial mediation model of SEC and PB in the association between PA and PS. Values are standardized path coefficients (β). PA = physical activity; SEC = social-emotional competence; PB = prosocial behavior; PS = perceived stress. *** *p* < 0.001.

Specifically, the estimated indirect association through Ind1 (PA → SEC → PS) was −0.0246 (95% CI [−0.030, −0.020]), accounting for 49.53% of the total indirect association. The estimated indirect association through Ind2 (PA → PB → PS) was −0.0161 (95% CI [−0.020, −0.013]), accounting for 32.52% of the total indirect association. The estimated serial indirect association through Ind3 (PA → SEC → PB → PS) was −0.0089 (95% CI [−0.011, −0.007]), accounting for 17.95% of the total indirect association. PARS-3 scores were right-skewed; however, a categorical sensitivity analysis using low, moderate, and high PA groups yielded results consistent with the continuous-score model (**Supplementary Material**). Taken together, these findings were consistent with a partial serial mediation pattern involving SEC and PB; however, the cross-sectional design does not permit conclusions regarding temporal or causal ordering.

## 4 Discussion

This study examined the relationships among PA, SEC, PB, and PS, with particular emphasis on the association between PA and PS among Chinese adolescents and the mediating roles of SEC and PB. PA, SEC, and PB were each negatively associated with adolescent PS. The data were statistically consistent with a serial indirect-association model involving SEC and PB. By integrating the psychological and social functions of PA within a unified framework based on COR theory, this study broadens the perspective from which the potential association between PA and adolescents’ stress experiences can be understood.

### 4.1 Association between PA and PS

PA was significantly negatively associated with PS among adolescents; students with higher PA generally reported lower PS, supporting H_1_. The cross-stressor adaptation hypothesis proposes that regular PA induces adaptive regulation of the autonomic nervous system and the hypothalamic-pituitary-adrenal axis and that this physiological adaptation transfers to psychosocial stress contexts, thereby attenuating psychophysiological stress responses and reducing PS (71, 72). Similar findings have been reported among working-age adults with overweight and psychological distress (73), suggesting that PA may represent an important protective behavior closely associated with PS.

Compared with university students and adults, adolescents are at a critical stage in which pubertal development, the reorganization of psychological and physical functions, and changing social roles intersect. Their emotion-regulation and stress-coping mechanisms remain under development, while their behavioral patterns retain substantial plasticity (74, 75). The regulatory effects of PA on physiological stress responses therefore provide a plausible explanation for its negative association with adolescent PS. Previous work has shown that adolescents who engage in PA at least two or three times per week report lower stress, consistent with the proposition that regular exercise promotes adaptation of the stress-response system and reduces stress-related physiological load (76). A five-year longitudinal study further showed that sustained participation in school sports during secondary school predicted lower PS in early adulthood, suggesting that long-term sport participation may reduce reactivity to subsequent stressors through cumulative physiological adaptation (77). Together, adolescents’ vulnerability to stress and behavioral plasticity provide a developmental basis for PA interventions, whereas adaptive regulation of the stress-response system provides a physiological rationale. These findings indicate an inverse association between PA and PS during adolescence, warranting longitudinal and intervention research in this developmental period. Schools, families, and communities could coordinate to expand adolescent PA opportunities. Whether such efforts reduce PS requires prospective or intervention evaluation.

### 4.2 Mediating role of SEC in the association between PA and PS

The indirect association between PA and PS involving SEC was statistically significant, consistent with H_2_. COR theory proposes that individuals are motivated to acquire, maintain, and protect resources used to cope with stress (9). Higher PA, higher SEC, and lower PS co-occurred in this sample. This pattern is compatible with, but does not establish, a resource-based explanation (78). A similar mediating pathway has been supported among adolescents with autism spectrum disorder (25), suggesting that SEC may represent a relevant psychological resource in different adolescent samples. By focusing on school-attending adolescents recruited from six secondary schools in Shandong and Henan Provinces and including PS as the outcome, the present study documents a comparable pattern of indirect associations in this specific regional school sample. Recent research suggests that team sports provide more stable psychosocial benefits than individual sports by strengthening peer belonging and social support through role coordination and peer interaction and by developing SEC that supports more effective coping (79, 80). The present findings indicate that the hypothesized indirect-association pattern is statistically compatible with data from Chinese adolescents experiencing substantial academic demands. They also establish a basis for longitudinal and intervention studies designed to examine temporal ordering and causality. Schools could consider team-based PA while prospectively evaluating its associations with SEC, stress-adjustment skills, and PS.

### 4.3 Mediating role of PB in the association between PA and PS

The indirect association between PA and PS involving PB was statistically significant, consistent with H_3_. PA, PB, social support, coping resources, and PS may be interrelated. Their temporal and causal ordering cannot be determined from these cross-sectional data (81; S. 42). Similar protective associations have been observed among university students exposed to negative life events or at risk of internet addiction (Q. 38), suggesting that PB may represent a psychosocial pathway linking PA to stress adaptation across populations. This study documents a comparable pattern of associations among school-attending adolescents facing substantial academic pressure. The educational and socializing value of PA may extend beyond improving interpersonal interaction. In this sample, PB was associated with both PA and PS and may partly characterize their statistical association (S. 42, 82). Although previous studies have examined PB and stress appraisal, many have adopted relatively fragmented variable frameworks (83). Experience-sampling and diary studies have linked PB to lower perceived stress among adults but have not incorporated PA, limiting their ability to explain the stress-buffering pathway of PB in sport contexts (84, 85). Using a sample of school-attending adolescents recruited from six secondary schools in Shandong and Henan Provinces, the present study integrates PB into a model linking PA with PS and provides evidence from this specific regional and cultural context. Such initiatives may provide opportunities for PB and social interaction. Their effects on interpersonal resources, group belonging, coping capacity, and PS require prospective evaluation.

### 4.4 Serial mediating roles of SEC and PB in the association between PA and PS

The serial indirect association between PA and PS involving SEC and PB was statistically significant, consistent with H_4_. COR theory proposes that individuals are motivated to acquire, maintain, and accumulate important resources, whereas resource scarcity and threats of resource loss are fundamental sources of stress (9). PA, SEC, and PB followed the prespecified serial indirect-association model. These cross-sectional data do not establish temporal ordering among the variables. PB, relational resources, and lower PS may be interrelated, but their temporal and causal ordering remains uncertain. Components of this serial pathway have also received support among university students and adults (85, 86), providing preliminary evidence of applicability across populations.

SEC and PB represent an internal psychological capacity and an overt social behavior, respectively. The observed associations were consistent with the prespecified ordering of PA, SEC, PB, and PS, but did not establish a progressive or temporal pathway. Longitudinal and cross-sectional studies have consistently linked SEC and its core components to adolescent PB. Self-regulation predicts subsequent PB, and dimensions of SEC and distinct self-regulation profiles are associated with both the level and developmental trajectory of PB (53, 87, 88). A school-based pilot study found that yoga enhanced adolescents’ SEC, possibly by increasing mind-body awareness and emotion regulation (89). A 12-week exercise intervention further indicated that collaborative tasks and values-based guidance in basketball allowed adolescents to experience teamwork and responsibility toward shared goals, thereby providing an experiential context for the development of PB (59). Both SEC and PB constitute positive developmental resources and were significantly negatively associated with PS. The pattern is consistent with interrelations among PA, emotional intelligence, social support, self-esteem, and PS, without establishing a temporal sequence (90; S. 42, 91). The present study evaluated a prespecified indirect-association model linking PA, SEC, PB, and PS. The cross-sectional design does not establish progression from internal capacity to overt behavior.

## 5 Limitations and future directions

This study has three principal limitations. First, the cross-sectional design and single-time-point assessment preclude conclusions about temporal ordering or causality and cannot capture potential long-term patterns of association. Reverse or reciprocal associations therefore cannot be excluded; for example, adolescents with higher PS may engage in less PA or report lower PB. Accordingly, the prespecified PA → SEC → PB → PS ordering should be interpreted as a statistical indirect-association model rather than a temporal pathway. Future research should use longitudinal follow-up, cross-lagged panel analysis, or randomized controlled trials to examine directionality and causal or dynamic relationships among these variables.

Second, the study relied primarily on self-report measures and may therefore be susceptible to recall bias, common method bias, and social desirability. Future studies could incorporate objective measures, including electroencephalography and heart-rate monitoring, and use wearable devices to record PA in real time. Behavioral observation could also be included to establish a multi-informant, multimethod assessment framework and improve the objectivity of the data and the reliability of the conclusions.

Finally, participants were recruited from six junior and senior secondary schools in inland cities of Shandong and Henan provinces, resulting in limited representation of adolescents from economically developed coastal areas, remote regions, and areas with large ethnic minority populations. Differences in regional culture, urban–rural context, school type, and family socioeconomic background were not fully examined, which may limit the external validity and generalizability of the findings. Future research should recruit school-attending adolescents from more geographically and socioeconomically diverse settings, including coastal and inland regions, urban and rural areas, remote communities, different school types, and various grade levels. Left-behind children, migrant children, and students experiencing high academic stress should also be considered. Multiple theoretical perspectives could also be integrated to examine differences by PA type, intensity, frequency, and organizational format and to clarify the psychological, behavioral, and physiological mechanisms linking PA to adolescent PS.

## 6 Conclusion

This study identified an inverse association between PA and PS and serial indirect associations involving SEC and PB. These findings describe a statistical pattern rather than causal mechanisms. In practice, schools should integrate the development of SEC and the promotion of PB into physical education and mental health education. The findings may inform stress interventions and mental health promotion for adolescents and provide useful guidance for policymakers, educators, and public health stakeholders. The findings support prospective evaluation of PA programs and resource-focused mental health strategies rather than conclusions about their effectiveness in reducing adolescent stress.

## Data Availability

The raw data supporting the conclusions of this article will be made available by the authors, without undue reservation.
