# Supplementary material for: The association between physical activity and perceived stress among adolescents: the serial mediating roles of social-emotional competence and prosocial behavior
Source: Front Psychiatry. 2026 Sep 16;17:1947638. doi: 10.3389/fpsyt.2026.1947638 (PMC13623936; doi:10.3389/fpsyt.2026.1947638)
Supplement: Supplementary file 1 [file Table1.docx]

**Supplementary Results**

**1) PARS-3 score distribution.**

The mean PARS-3 score was 30.18 (SD = 24.56), the median was 24 (IQR = 12–48), and the range was 0–100. The distribution was right-skewed (skewness = 1.049, excess kurtosis = 0.477); 111 participants scored 0 (3.86%). Participants with low, moderate, and high levels of physical activity accounted for 44.97%,28.30%, and 26.73%, respectively (Table S1).

**Table S1 | Distribution characteristics of the PARS-3 score（n=2,873）**

| **Indicator** | **Results** |
| --- | --- |
| Mean | 30.18 |
| Standard Deviation | 24.56 |
| Median, | 24 |
| Interquartile Range | 12-48 |
| Minimum–Maximum | 0-100 |
| Skewness | 1.049 |
| Excess peakedness | 0.477 |
| Exact zero value | 111（3.86%） |
| Low activity（≤19） | 1,292（44.97%） |
| Central Activity（20-42） | 813（28.30%） |
| High Activity（>42） | 768（26.73%） |

**2) Physical activity level sensitivity analysis.**

The categorical sensitivity analysis revealed that the direction of the direct effect, total effect, and the three specific indirect effects for the moderate-and high-activity groups compared to the low-activity group was consistent with that observed in the continuous variable model; moreover, all 95% bootstrap confidence intervals did not contain 0 (Table S2).

**Table S2 | Standardized effects of the Physical Activity Level Model (with the low-activity group as the reference)**

| **Path** | **Moderate-activity group vs. low-activity group, β [95% CI]** | **High-activity group vs. low-activity group, β [95% CI]** |
| --- | --- | --- |
| X→M1 (a₁) | 0.6004 [0.5235, 0.6792] | 0.9802 [0.9030, 1.0606] |
| X→M2, adjusted for M1 (a₂) | 0.3079 [0.2299, 0.3821] | 0.6068 [0.5262, 0.6906] |
| X → Y: direct association (c′) | -0.2522 [-0.3339, -0.1699] | -0.4980 [-0.5820, -0.4163] |
| X → Y: total association (c) | -0.4941 [-0.5764, -0.4116] | -0.9152 [-0.9952, -0.8363] |
| Indirect association via M1 (a₁b₁) | -0.1261 [-0.1570, -0.0990] | -0.2059 [-0.2486, -0.1651] |
| Indirect association via M2 (a₂b₂) | -0.0660 [-0.0867, -0.0468] | -0.1301 [-0.1590, -0.1034] |
| Serial indirect association via M1 and M2 (a₁d₂₁b₂) | -0.0497 [-0.0619, -0.0390] | -0.0811 [-0.0983, -0.0650] |
| Total indirect association | -0.2418 [-0.2812, -0.2057] | -0.4171 [-0.4690, -0.3673] |

Note. The low-activity group was used as the reference category. β denotes the standardized coefficient, and values in brackets are 95% confidence intervals. M1 represents social-emotional competence (SEC), M2 represents prosocial behavior (PB), and Y represents perceived stress (PS). All models were adjusted for sex, age, grade, birthplace, only-child status, and boarding status. Confidence intervals for the indirect associations were estimated using 5,000 bootstrap resamples.
